# Laboratory‐based surveillance of *Candida auris* in Colombia, 2016–2020

**DOI:** 10.1111/myc.13390

**Published:** 2021-12-03

**Authors:** Patricia Escandón, Diego H. Cáceres, Diana Lizarazo, Shawn R. Lockhart, Meghan Lyman, Carolina Duarte

**Affiliations:** ^1^ Grupo de Microbiología Instituto Nacional de Salud Bogotá Colombia; ^2^ Centers for Disease Control and Prevention (CDC) Atlanta Georgia USA; ^3^ Center of Expertise in Mycology Radboudumc/CWZ Nijmegen The Netherlands

**Keywords:** bloodstream infection, *Candida auris*, Colombia, COVID‐19, laboratory surveillance, SARS‐CoV‐2

## Abstract

**Background:**

Since the first report of *Candida auris* in 2016, the Colombian Instituto Nacional de Salud (INS) has implemented a national surveillance of the emerging multidrug‐resistant fungus.

**Objectives:**

This report summarises the findings of this laboratory‐based surveillance from March 2016 to December 2020.

**Results:**

A total of 1720 *C. auris* cases were identified, including 393 (23%) colonisation cases and 1327 (77%) clinical cases. Cases were reported in 20 of 32 (62%) departments of Colombia and involved hospitals from 33 cities. The median age of patients was 34 years; 317 (18%) cases were children under 16 years, 54% were male. The peak number of cases was observed in 2019 (*n* = 541). In 2020, 379 (94%) of 404 cases reported were clinical cases, including 225 bloodstream infections (BSI) and 154 non‐BSI. Among the 404 cases reported in 2020, severe COVID‐19 was reported in 122 (30%). Antifungal susceptibility was tested in 379 isolates. Using CDC tentative breakpoints for resistance, 35% of isolates were fluconazole resistant, 33% were amphotericin B resistant, and 0.3% isolates were anidulafungin resistant, 12% were multidrug resistant, and no pan‐resistant isolates were identified.

**Conclusion:**

For five years of surveillance, we observed an increase in the number and geographic spread of clinical cases and an increase in fluconazole resistance. These observations emphasise the need for improved measures to mitigate spread.

## 1 INTRODUCTION

In Colombia, surveillance of the multidrug‐resistant yeast *Candida auris* began in 2016, with the identification of an unusual number of bloodstream infections caused by *C haemulonii*, later confirmed by molecular methods as *C. auris*. Establishing local spread of this emerging pathogen in Colombia prompted the public health authorities to issue a national alert on the emergence of *C. auris*, leading to the ongoing surveillance which has revealed a continuous increase in cases.^1^ The ability of this microorganism to colonise patients and persist on environmental surfaces contributes to *C. auris* outbreaks in healthcare facilities, especially in intensive care units (ICU).^2^, ^3^ The aim of this report is to summarise the main results of national laboratory surveillance of *C. auris* in Colombia from 2016 to 2020.

## 2 MATERIALS AND METHODS

In 2016, the Colombian Instituto Nacional de Salud (INS) released an epidemiological alert on *Candida auris*. In this alert, INS requested that public health laboratories in the Colombian territory send all suspected or confirmed *C. auris* isolates to the mycology reference laboratory at the INS in Bogotá, Colombia for species identification and antifungal susceptibility testing (AFST).^1^ Confirmation of species identification was performed using Biotyper MALDI‐TOF (Bruker^TM^, Billerica, MA, USA). AFST was done following the methodology described by the Clinical and Laboratory Standards Institute^4^ and/or the Sensititre antimicrobial susceptibility testing system (Thermo Scientific^TM^, Waltham, MA, USA). For interpretation of AFST results, we calculated the minimum inhibitory concentration (MIC) 50 (MIC_50_) and 90 (MIC_90_), and we also used tentative MIC breakpoints suggested by CDC for *C. auris*, where resistance is defined as ≥32 µg/ml for fluconazole, ≥2 µg/ml for amphotericin B, ≥4 µg/ml for anidulafungin and micafungin and ≥2 µg/ml for caspofungin.^5^ AFST was performed only where supplies were available.

Isolates were referred to the national reference laboratory, using a standardised submission form that collected basic demographic and clinical information from cases. For the purpose of this report, *C. auris* cases were classified according to the *Standardized Case Definition for Candida auris clinical and colonization/screening cases*, developed by the Council of State and Territorial Epidemiologist (CSTE) of the United States of America.^6^ A *C. auris* colonisation case was defined as a person with confirmatory laboratory evidence from a swab collected for the purpose of screening for colonisation regardless of site swabbed.^6^ A clinical case was defined as a person with confirmatory laboratory evidence from a clinical specimen collected for the purpose of diagnosing or treating disease in the normal course of care.^6^ We further classified clinical cases into two categories according to isolate source: bloodstream infection (BSI) clinical cases and non‐BSI clinical cases.

## 3 RESULTS

**FIGURE 1:**
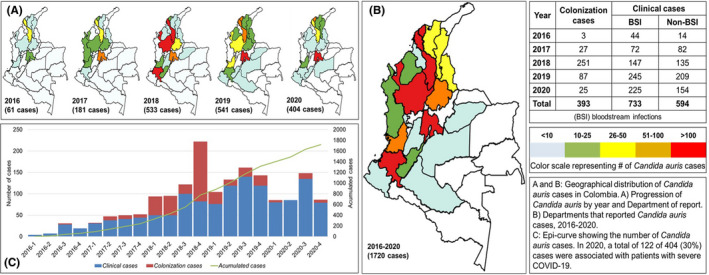
Confirmed cases of *Candida auris* in Colombia, 2016–2020

From 2016 to 2020, 1720 *C. auris* cases were identified, including 393 (23%) colonisation cases and 1327 (77%) clinical cases; 733 [55%] BSIs and 594 [45%] non‐BSIs. For non‐BSIs, specimens of *C. auris* were isolated from genital‐urinary tract: *n* = 248, catheter: *n* = 95, respiratory specimens: *n* = 88, gastrointestinal specimens: *n* = 71, skin lesions: *n* = 21, bone: *n* = 18, unspecified tissue: *n* = 31, paranasal/ears/nose/mouth: *n* = 9, unspecified fluids: *n* = 7 and central nervous system: *n* = 6. Non‐BSIs specimens were collected for the purposes of diagnosing or treating disease in the normal course of care. The highest number of total cases (*n* = 541) and clinical cases (*n* = 454, 84%) was observed in 2019 (Figure 1). The largest number of colonisation cases (*n* = 251) occurred in 2018, likely related to the *C. auris* colonisation screening efforts being conducted as part of outbreak investigations (Figure 1). In 2019 and 2020, a decrease in the number of colonisation cases was observed, with only 87 cases in 2019 and 25 cases in 2020. During 2020, 94% of cases reported were clinical cases (379 of 404 cases), more than half of which were BSIs (*n* = 225, 59%) (Figure 1). Cases were reported in 20 of 32 (62%) departments of Colombia (country subdivisions) and involved hospitals from 33 cities. *C. auris* cases were initially reported in the northeast region of the country (Caribbean region) ^7^, but by 2020, all departments of the Colombian west coast (Pacific coast) and the central region of the country (Andes region) had experienced cases (Figure 1).

From the total number of cases, 930 (54%) were from male patients. The median age of patients was 34 years. Children under 16 years represented 18% (*n* = 317) of total cases, the youngest being a 1‐day‐old male patient with a BSI. Patients over 70 years old accounted for 20% (*n* = 348) of cases, with the oldest patient being 97 years old. Among the 404 *C. auris* cases reported in 2020, severe COVID‐19 was reported in 122 (30%). Of these 122 cases with COVID‐19, 113 (93%) were clinical cases, and 66% of those were BSIs (*n* = 75).

Antifungal susceptibility was done in isolates obtained from clinical cases, in addition, testing was not frequently performed, and frequency varied by antifungal. For azole medications, 404 (24%) isolates were tested for fluconazole susceptibility and 225 (13%) for voriconazole susceptibility. For echinocandins, 381 (22%) isolates were tested for susceptibility to anidulafungin, 83 (5%) to caspofungin and 83 (5%) to micafungin. Amphotericin susceptibility testing was performed for 399 (23%) isolates. There were 379 isolates, 305 from BSI and 74 non‐BSI isolates, tested for all three classes (ie fluconazole, amphotericin B and anidulafungin, Table 1). Using CDC tentative breakpoints, 131 (35%) of these 379 isolates were fluconazole resistant, 125 (33%) were amphotericin B resistant, and one (0.3%) isolate was echinocandin resistant. Forty‐five of 379 isolates (12%) were multidrug resistant (44 fluconazole/amphotericin B resistant, and one fluconazole/anidulafungin resistant); no pan‐resistant isolates were identified. The MIC_50_ for fluconazole changed over time; 4 µg/ml in 2016, 8 µg/ml in 2018, 4 µg/ml in 2019 and 32 µg/ml in 2020 (Table 2). This pattern was not observed for the other antifungals. No AFST data were available for isolates collected in 2017.

**TABLE 1:** Summary of MIC_50_ and MIC_90_ in µg/ml for antifungal drugs against Colombian *C. auris* isolates, March 2016 to December 2020

Drug	Fluconazole (*n* = 404)	Voriconazole (*n* = 225)	Amphotericin B (*n* = 399)	Anidulafungin (*n* = 381)	Caspofungin (*n* = 84)	Micafungin (*n* = 83)
MIC_50_	4	0.5	1	0.125	0.06	0.06
MIC_90_	64	4	4	0.25	0.38	1

Abbreviations: AFST, antifungal susceptibility testing; MIC, Minimum inhibitory concentration; MIC_50_, minimum inhibitory concentration 50; MIC_90_, minimum inhibitory concentration 90; number of isolates tested (*n*).

**TABLE 2:** AFST/time analysis for fluconazole MIC_50_ and MIC_90_ in µg/ml on Colombian *C. auris* isolates

Year	2016 (*n* = 21)	2018 (*n* = 15)	2019 (*n* = 183)	2020 (*n* = 185)
MIC_50_	4	8	4	32
MIC_90_	32	32	32	64

Abbreviations: AFST, antifungal susceptibility testing; MIC, Minimum inhibitory concentration; MIC_50_, minimum inhibitory concentration 50; MIC_90_, minimum inhibitory concentration 90; number of isolates tested (*n*).

## 4 DISCUSSION

Five years of surveillance detected spread of *Candida auris* in the Colombian territory with an increase in the number of overall and clinical cases, geographic area reporting cases and fluconazole resistance. AFST indicated that about two thirds of isolates were resistant to at least one antifungal, one‐third resistant to fluconazole, and the other third were resistant to amphotericin B. Multidrug resistance, mostly fluconazole/amphotericin B, was observed in about one of ten isolates tested. In comparison with other regions of the world like India, Pakistan, South Africa or neighbour countries like Venezuela or Panama, fluconazole resistant and multidrug resistant is lower. In Colombia, fluconazole is not used to treat invasive *Candida* infections, but the recent increase in fluconazole resistant is concerning.^8^, ^9^, ^10^ The factors responsible for the increase of fluconazole resistance are unknown, but there could be multiple causes. An increase in transmission of resistant strains or the introduction of new *C. auris* clades in Colombian territory could be responsible, further investigation of molecular diversity of Colombian *C. auris* isolates is needed.

To contain the spread of *C. auris*, the Instituto Nacional de Salud de Colombia INS released national clinical alerts and has mandated *C. auris* reporting in the Colombian territory.^1^ Although the overall number of clinical cases have increased, the percentage of colonisation cases decreased in recent years. The most likely explanation for this discrepancy between the increase of clinical cases and decrease of colonisationcases is the modification in the national *C. auris* guidelines introduced in 2019 that recommended excluding colonisationcases not linked to an outbreak from the national *C. auris* case count. In addition, there were delays in case notification due to the COVID‐19 pandemic which overstretched resources and resulted in an underestimate of cases in 2020. In addition, AFST was not performed as routine testing on samples collected from colonised patients. Further efforts to build AFST capacity and investigate the resistance patterns are needed in Colombia to understand the factors related to this increase and understand characteristics and outcomes of cases.

The main limitation of the laboratory‐based surveillance in Colombia is the lack of clinical and epidemiological data from cases, the underestimation of colonised cases results of changes in surveillance since 2019 and the lack of genotyping of isolates. Despite these limitations, data generated by this system are essential for guiding investigation and response activities, including infection control practices which are essential to prevent the spread of *C. auris* and other resistant pathogens. The observed increase in *C*. *auris* cases in healthcare facilities across Colombia reinforces the importance of mandatory public health reporting to continue surveillance for this and other resistant fungal pathogens.

## CONFLICT OF INTEREST

All authors report no potential conflicts of interest. The findings and conclusions in this report are those of the authors and do not necessarily represent the official position of the funding agencies.

## AUTHOR CONTRIBUTION

**Patricia Escandon:** Conceptualization (equal); Data curation (equal); Formal analysis (equal); Funding acquisition (equal); Investigation (equal); Methodology (equal); Project administration (equal); Resources (equal); Software (equal); Writing‐original draft (equal); Writing‐review & editing (equal). **Diego H Caceres:** Conceptualization (equal); Data curation (equal); Formal analysis (equal); Investigation (equal); Methodology (equal); Resources (equal); Software (equal); Supervision (equal); Validation (equal); Writing‐original draft (equal); Writing‐review & editing (equal). **Diana Lizarazo:** Investigation (equal); Writing‐review & editing (equal). **Shawn Lockhart:** Formal analysis (equal); Investigation (equal); Supervision (equal); Validation (equal); Writing‐original draft (equal); Writing‐review & editing (equal). **Meghan Marie Lyman:** Formal analysis (equal); Investigation (equal); Methodology (equal); Supervision (equal); Writing‐original draft (equal); Writing‐review & editing (equal). **Carolina Duarte Valderrama:** Investigation (equal); Project administration (equal); Supervision (equal); Writing‐original draft (equal); Writing‐review & editing (equal).
